# A systematic analysis of orphan cyclins reveals CNTD2 as a new oncogenic driver in lung cancer

**DOI:** 10.1038/s41598-017-10770-8

**Published:** 2017-08-31

**Authors:** L. Gasa, A. Sanchez-Botet, E. Quandt, S. Hernández-Ortega, J. Jiménez, M. A. Carrasco-García, S. Simonetti, S. J. Kron, M. P. Ribeiro, E. Nadal, A. Villanueva, J. Clotet

**Affiliations:** 1 Faculty of Medicine and Health Sciences, Universitat Internacional de Catalunya, Barcelona, Spain; 2 Pathology Department, Hospital Universitari General de Catalunya, Sant Cugat del Vallès, Barcelona, Spain; 3 Department of Molecular Genetics and Cell Biology, The University of Chicago, Chicago, USA; 4 Department of Medical Oncology and Program in Molecular Mechanisms and Experimental Therapeutics in Oncology, Catalan Institute of Oncology (ICO) Bellvitge Biomedical Research Institute (IDIBELL), L’Hospitalet del Llobregat, Barcelona, Spain; 5 Chemoresistance and Predictive Factors Group, Program Against Cancer Therapeutic Resistance (ProCURE), Catalan Institute of Oncology (ICO) Bellvitge Biomedical Research Institute (IDIBELL), L’Hospitalet del Llobregat, Barcelona, Spain

## Abstract

As lung cancer has increased to the most common cause of cancer death worldwide, prognostic biomarkers and effective targeted treatments remain lacking despite advances based on patients’ stratification. Multiple core cyclins, best known as drivers of cell proliferation, are commonly deregulated in lung cancer where they may serve as oncogenes. The recent expansion of the cyclin family raises the question whether new members might play oncogenic roles as well. Here, we investigated the protein levels of eight atypical cyclins in lung cancer cell lines and formalin-fixed and paraffin-embedded (FFPE) human tumors, as well as their functional role in lung cancer cells. Of the new cyclins evaluated, CNTD2 was significantly overexpressed in lung cancer compared to adjacent normal tissue, and exhibited a predominant nuclear location. CNTD2 overexpression increased lung cancer cell viability, Ki-67 intensity and clonogenicity and promoted lung cancer cell migration. Accordingly, CNTD2 enhanced tumor growth *in vivo* on A549 xenograft models. Finally, the analysis of gene expression data revealed a high correlation between elevated levels of CNTD2 and decreased overall survival in lung cancer patients. Our results reveal CNTD2 as a new oncogenic driver in lung cancer, suggesting value as a prognostic biomarker and therapeutic target in this disease.

## Introduction

Lung cancer is expected to be responsible for over 275,000 deaths in the European Union in the year of 2016, representing more than 20% of total cancer mortality^1^. Around 80% of lung cancers are non-small-cell lung cancers (NSCLC), whose management remains challenging despite recent advances based on tumor genetic stratification using relevant biomarkers, such as EGFR, ALK, ROS-1, MET and KRAS^2^. While surgery or radiotherapy can cure early stage, localized tumors, high rates of local and distant relapse still occur^3^. Even then, the majority of NSCLC patients are not candidates for surgery due to their advanced or metastatic disease at diagnosis^4^. Despite progress in targeted therapies, most NSCLCs do not present known targetable mutations. Only one in five NSCLC patients respond to the approved checkpoint blockade immunotherapies^5^. Therefore, a deeper understanding of the molecular alterations underlying lung cancer development and progression may contribute not only to the identification of therapeutic targets, but also to the establishment of new prognostic and predictive biomarkers.

Loss of growth control is a hallmark of cancer and a common target of cancer therapeutics. Progression through the cell cycle is regulated by members of the cyclin-dependent kinase family (CDKs), a group of highly conserved serine/threonine kinases that must associate with cyclin proteins to phosphorylate their substrates^6^. Cyclin binding provides each CDK with targeting domains that mediate substrate binding and determine subcellular localization, which in turn determine biological specificity. As such, specific cyclin-CDK complexes are associated with each major transition in the cell cycle. Many cancers display inappropriate expression of the canonical cell cycle cyclins. Here, they may serve as oncogenes by activating cell cycle CDKs to support deregulated cancer cell proliferation. In the case of lung cancer, upregulation of cyclin B1, which binds CDK1 to drive mitosis, was linked to a poor prognosis in NSCLC^7^. Likewise, decreased overall survival was observed in tumors overexpressing cyclin D1 which activates CDKs 4 and 6 in G1 phase^8^. In turn, EGFR inhibition downregulates cyclin D1, suggesting loss of cyclin-CDK activity may mediate effects of EGFR inhibitors^9^. Small molecules such as the approved agent palbociclib (Ibrance, Pfizer) and other cyclin D-CDK4/6 inhibitors demonstrate activity in multiple cancers including NSCLC, validating CDKs as therapeutic targets^10, 11^.

Most studies of lung cancer initiation and progression have limited their analysis to the canonical cyclins such as cyclin D, ignoring many other expressed genes that encode a characteristic “cyclin box”, the ~150 residue domain that determines CDK binding^12, 13^. While some of these “new” candidate cyclins are now known to bind the non-cell cycle CDKs that control transcription^14^, others remain “orphans”, where their CDK partner(s) remain to be identified^15^. Based on results from genetic model systems, some of these orphan cyclins are likely to serve regulatory roles in cell proliferation. Strikingly, the possible roles of non-canonical cyclins, including the orphan cyclins, remain largely unexplored in malignancies including lung cancer, and may lead to the development of innovative therapeutic strategies that complement cisplatin-based chemotherapy or CDK inhibitors.

Notably, previous analyses of altered gene expression in lung cancer have not identified orphan cyclins and being overexpressed or silenced. Nonetheless, the weak correspondence between the transcriptome and proteome often observed in normal cells also presents significant challenges in lung cancer^16^. The discordance likely reflects the importance of post-transcriptional control in determining cellular protein levels^17, 18^. Given the many post-translational mechanisms that determine the abundance of canonical cyclins, we considered that a direct assessment of protein levels would be needed to detect altered expression of the orphan cyclins.

In the present work, we used immunodetection to probe expression of eight orphan cyclins in human lung cancer cell lines, as well as in resected NSCLC tumors and identified CNTD2 as commonly overexpressed in lung cancer. Studies in lung cancer cell lines and xenograft mouse models as well as patient data suggested altered expression of CNTD2 may have functional significance. Our work suggests that non-canonical cyclins such as CNTD2 have the potential to serve as oncogenes in lung cancer and have potential as prognostic markers in NSCLC.

## Results

### The expression of CNTD2 and CCNI is increased in lung cancer tissues

**Figure 1:**
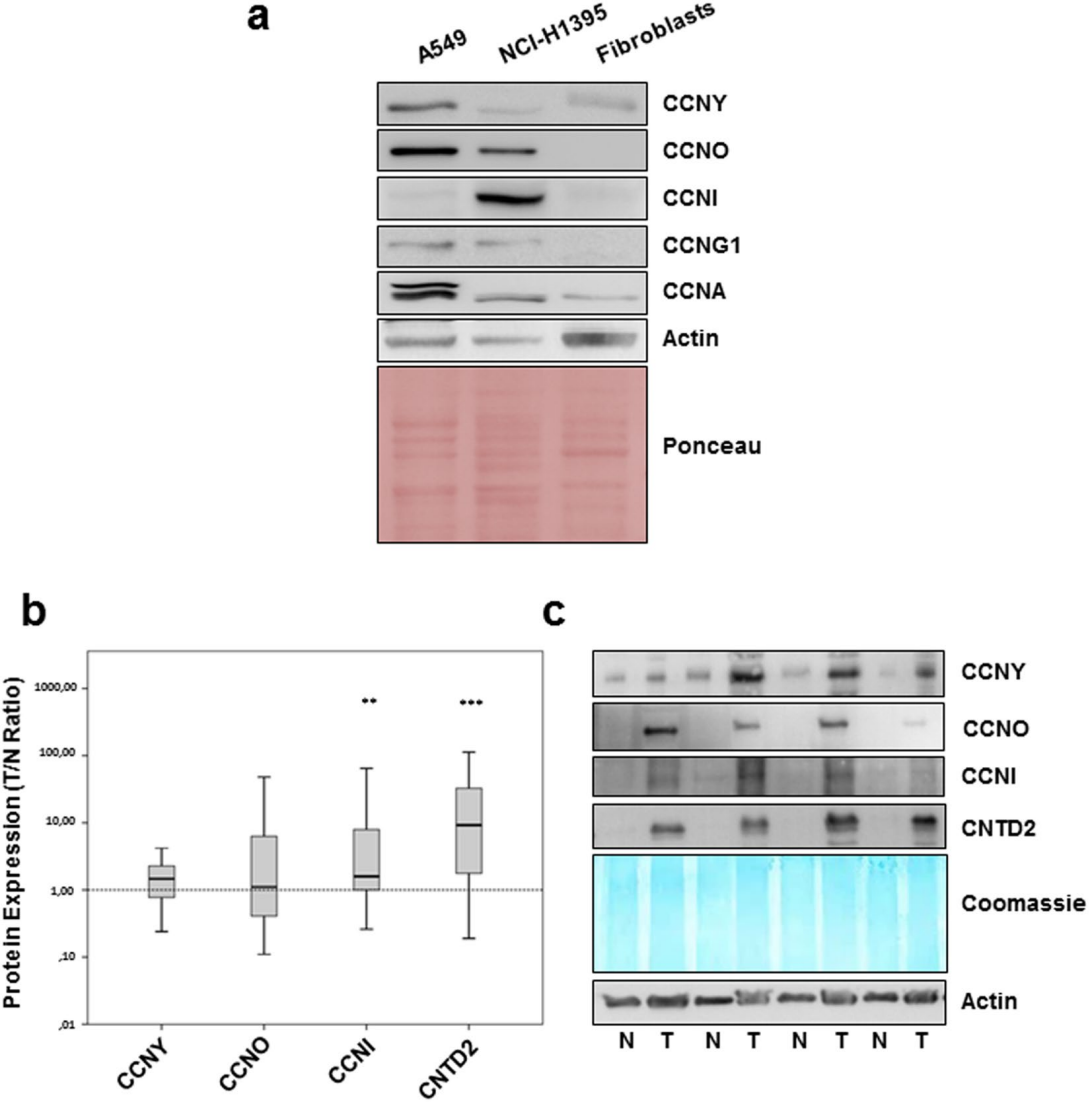
CNTD2 and CCNI are overexpressed in human lung cancer tissues. The expression of orphan cyclins was evaluated by western blot analysis. (**a**) Representative images of the expression of orphan cyclins in human lung adenocarcinoma cell lines, A549 and NCI-H1395, and in normal fibroblasts. (**b**) The protein expression of orphan cyclins in human FFPE lung cancer tissues and paired-adjacent non-tumor lung tissue is presented as box plots of the expression ratio between tumor and normal lung tissues (T/N ratio), where the whiskers indicate the range of the data and the horizontal bars represent the median (n = 43). **P < 0.01, ***P < 0.001 vs normal tissues, Wilcoxon test. (**c**) Representative images of the orphan cyclins protein expression in human normal (N) and tumor (T) FFPE lung tissue.

To investigate the potential role of orphan cyclins in lung cancer, the expression of CCNG1, CCNG2, CCNI, CCNO, CCNY, CNTD1, CNTD2 and SPY1 was investigated in two lung adenocarcinoma cell lines, A549 and NCI-H1395, relative to a primary culture of human fibroblasts by Western blot (Fig. 1a) using antibodies validated against recombinant proteins (Supplementary Fig. S1). Examining expression of the canonical CCNA, as a positive control, revealed CCNA is up-regulated in both cancer cell lines, and particularly in A549 cells, relative to the normal fibroblasts (Fig. 1a). Expression of CNTD1, CNTD2 CCNG2, or SPY1 was undetectable in the tumor or normal cells (Supplementary Fig. S2a). However, compared to normal fibroblasts, expression of CCNY, CCNO and CCNG1 was higher in A549 cells while levels of CCNO, CCNG1 and CCNI were higher in NCI-H1395 cells (Fig. 1a). These results indicate that the expression of some orphan cyclins might be misregulated in lung cancer and that the pattern of their expression is cell type-specific. These observations were further supported by comparing the expression of orphan cyclins in NCI-H1395 cells and in the human lung fibroblast MRC-5 cell line (Supplementary Fig. S2b).

In order to further investigate the orphan cyclin expression in lung cancer, Western blot analysis was performed in 43 formalin-fixed and paraffin-embedded (FFPE) human lung cancer samples and paired adjacent non-tumor lung tissue. Patient characteristics are summarized in Supplementary Table S1. The expression ratio between tumor and normal tissues (T/N ratio) was determined after normalization using Coomassie staining of the membrane. Median protein expression of CCNY, CCNO, CCNI, and CNTD2 was higher in lung cancer than normal lung tissue, but statistical significance was only found for CCNI and CNTD2 (Fig. 1b and c). The 17 kDa isoform of CNTD2 described by Uniprot was also expressed, but no difference was observed between normal and tumor tissue (data not shown).

**Figure 2:**
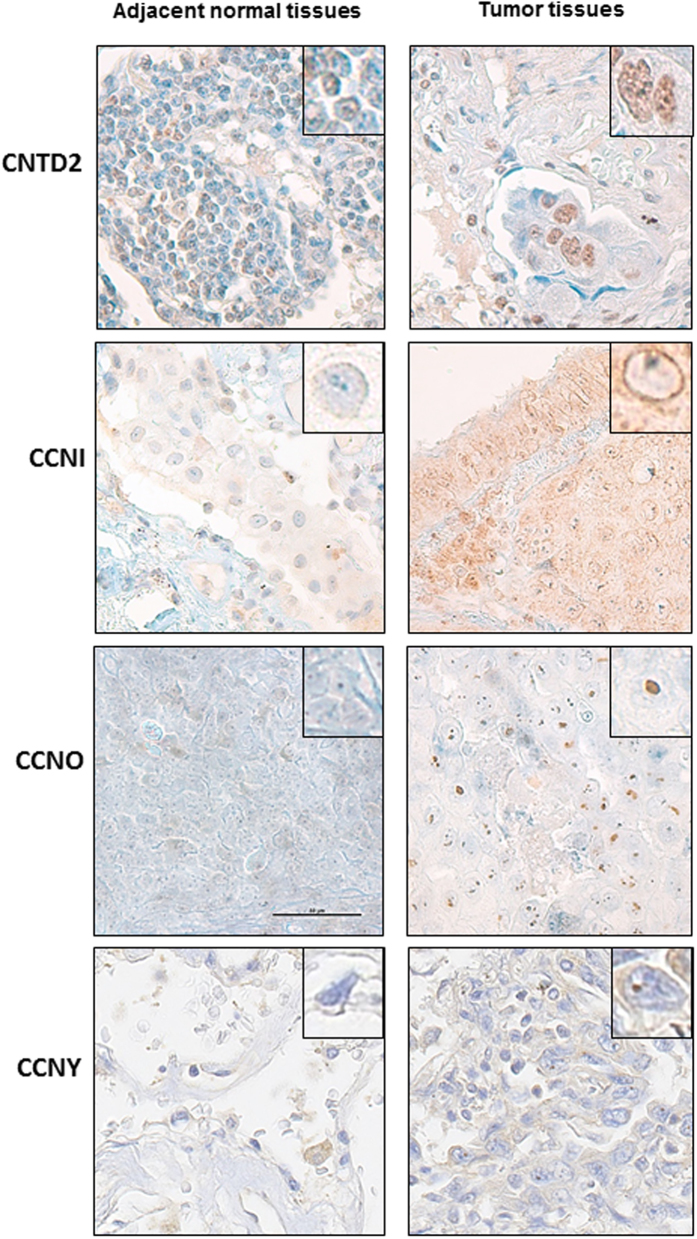
Cellular location of overexpressed orphan cyclins in human lung tissues. Representative images of the immunohistochemical staining of human lung adenocarcinoma samples with CNTD2, CCNI, CCNY and CCNO antibodies. Adjacent normal tissues were used as a control. Magnification 60x. The square reveals a detail of a representative cell with a magnification of 100x.

Confirming the results obtained of Western analysis, examining six pairs of lung cancer and paired adjacent normal tissue by immunohistochemistry demonstrated positive staining for CNTD2, CCNO and CCNI while adjacent normal lung was generally negative (Fig. 2). A weak signal of CCNY was detected in both tumor and normal tissues (Fig. 2). CNTD2 was predominantly localized to the nuclei of tumor cells, while the CCNI localized to the nuclear membrane and CCNO appeared concentrated in nucleoli.

### Overexpression of CNTD2 and CCNI enhances the proliferation of lung cancer cells

**Figure 3:**
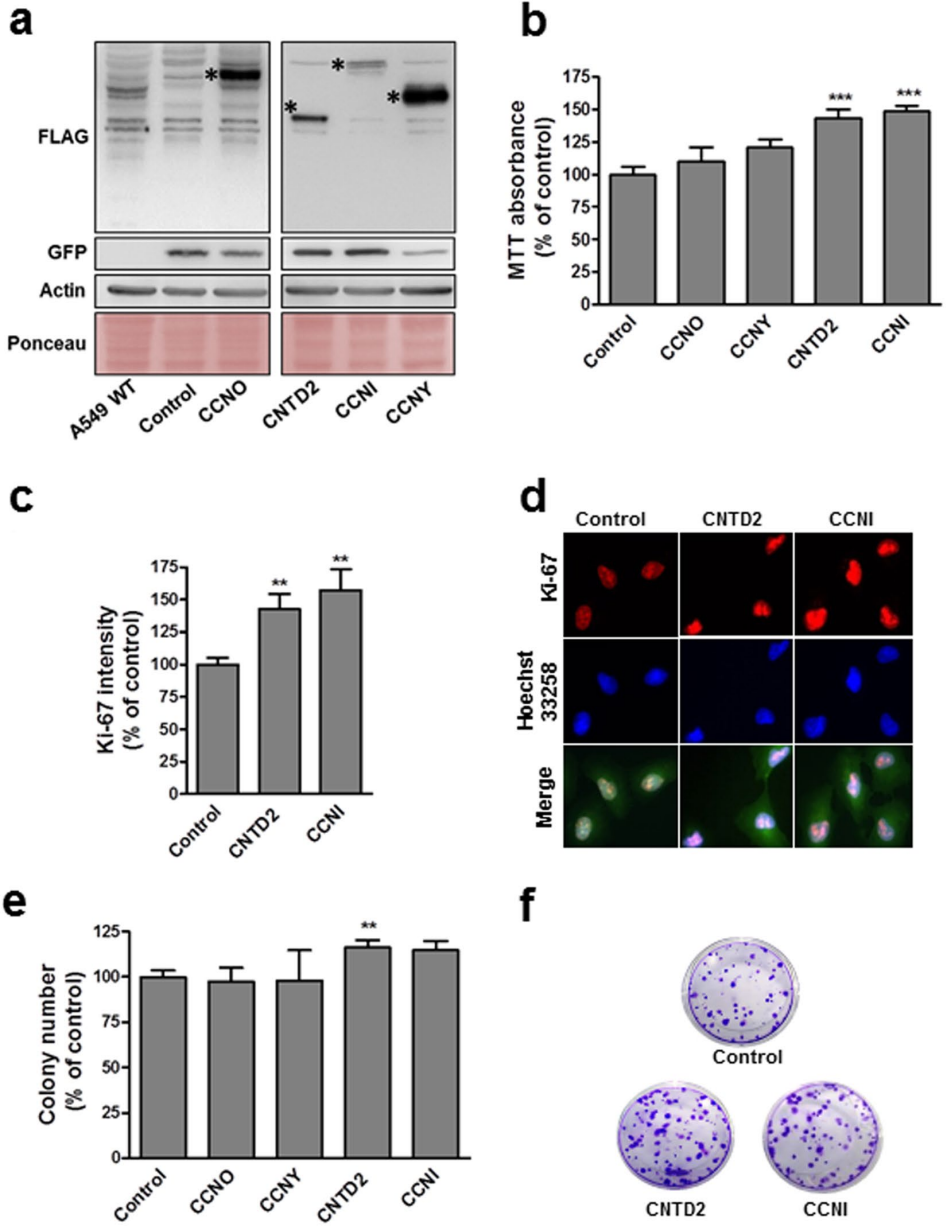
Overexpression of CCNI and CNTD2 increases the proliferation of A549 cells. A549 cells were infected with empty lentiviral vector (control) or with the indicated cyclin-overexpressing construct. (**a**) Western blot analysis confirmed cell infection (the asterisks show the expected band size). The flag indicates the protein expression level, the GFP the infection level and both the ponceau and the actin are used as loading controls. (**b**) A549 cell viability was evaluated by the MTT assay. Columns represent the mean ± SEM of five independent experiments performed in quadruplicates. ***P < 0.001 vs control, Mann-Whitney test. (**c**) The proliferation of A549 cells was evaluated by ki-67 immunostaining. Columns represent the mean ± SEM of four independent experiments. **P < 0.01 vs control, Mann-Whitney test. (**d**) Representative images of A549 cells immunostained for ki-67. Cell nuclei were counterstained with Hoechst 33258 and merged images are shown in the bottom row. (**e**) Efficiency of cell colony formation of A549 cells. Columns represent the mean ± SEM of four independent experiments performed in quintuplicates. **P < 0.01, vs control, Mann-Whitney test. (**f**) Representative images of the colony formation assay.

To examine potential roles for orphan cyclins in cancer cell growth or survival, A549 cells were transduced with empty lentiviral vector (control) or constructs expressing CCNI, CCNO, CCNY or CNTD2 (Fig. 3). Flow cytometry to detect GFP expression from lentivirus indicated an infection efficiency above 85% (data now shown) while Western blot confirmed overexpression (Fig. 3a). Cell viability evaluated by MTT assay 5 days after transduction revealed a significant impact of CCNI and CNTD2 overexpression on cell viability (Fig. 3b). Suggesting an effect on cell proliferation, A549 cells overexpressing CCNI and CNTD2 exhibited significantly higher Ki-67 staining (Fig. 3c and d). On the other hand, the expression of caspase-9 was monitored by western blot in A549 cells overexpressing CNTD2 and CCNI, and no significant alterations were found relative to control (Supplementary Fig. S3). Overexpression of CCNI and CNTD2 also increased clonogenicity of A549 cells, although statistical significance was only found for CNTD2 (Fig. 3e and f). Examining effects on cell survival, A549 cells overexpressing CCNI were also slightly less sensitive to cisplatin, gefitinib, and paclitaxel while overexpression of CNTD2 had little or no effect (Supplementary Fig. S4).

### The overexpression of CNTD2 promotes lung cancer cell migration

**Figure 4:**
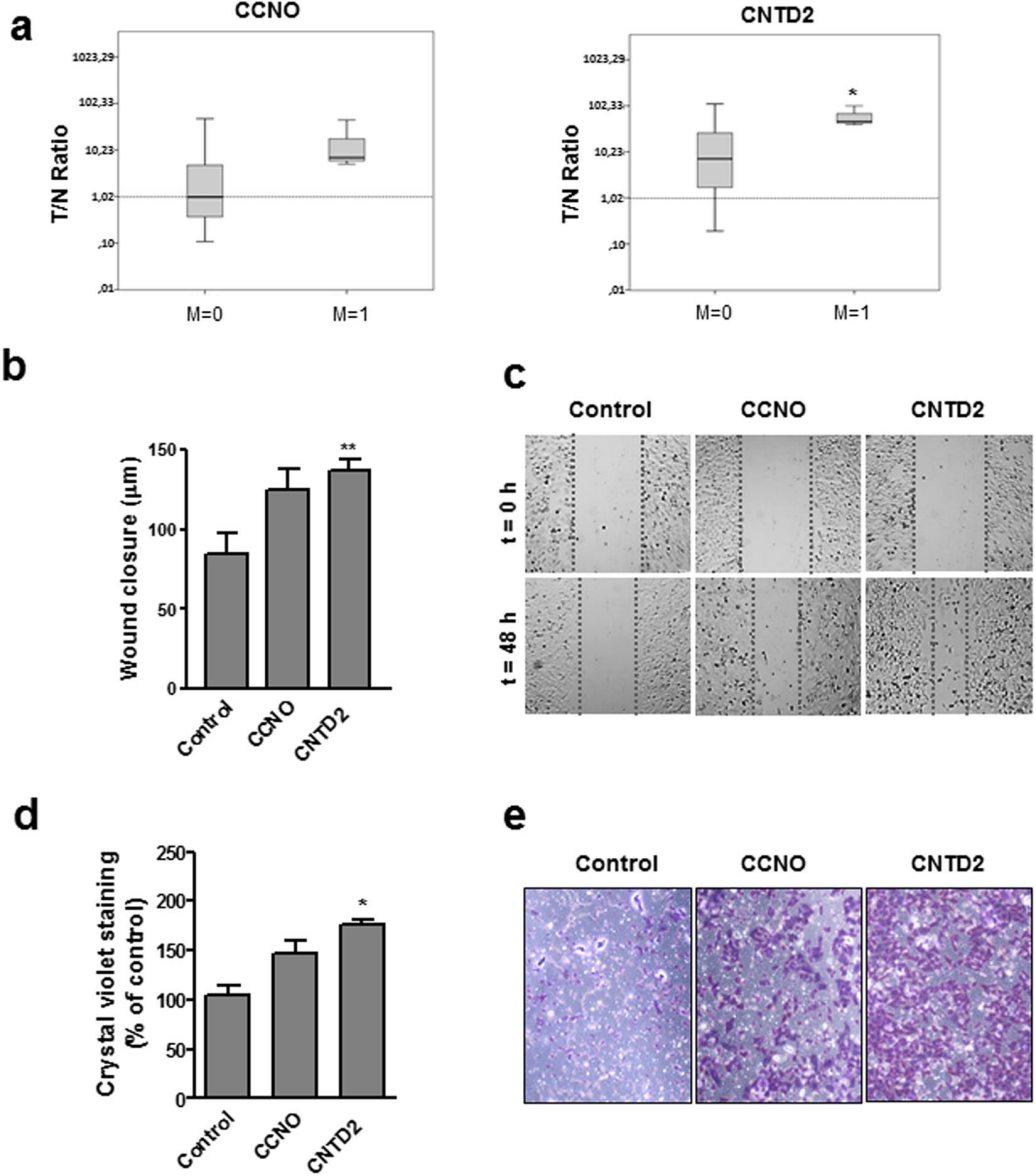
Overexpression of CNTD2 enhances the migration of A549 cells. (**a**) The T/N ratio of CCNO and CNTD2 was evaluated in FFPE lung tissue samples obtained from patients with (M1, n = 3) and without metastasis (M0, n = 40) and is represented as box plots. *P < 0.05, when compared to normal tissues, Mann-Whitney test. (**b**) The migration of A549 cells was evaluated by the wound assay and the results are expressed as wound closure in µm. The columns represent the mean ± SEM of three independent experiments performed in triplicates. **P < 0.01 vs control, Mann-Whitney test. (**c**) Representative images of the wound assay were acquired at 0 and 48 h; the dotted lines define the areas lacking cells. (**d**) A549 cell migration was monitored by the transwell assay and estimated by quantifying the crystal violet staining intensity measured as the absorbance at 595 nm. The columns represent the mean ± SEM of four independent experiments performed in triplicate. *P < 0.05 vs control, Mann-Whitney test. (**e**) Representative images of the transwell assay after crystal violet staining.

To explore potential links between orphan cyclin expression and metastasis, we determined the T/N ratio for each cyclin in tumors from patients with metastasis (M1) and without metastasis (M0). The T/N expression ratio of CCNO and CNTD2 was higher among patients with distant metastasis, but statistical significance was only found for CNTD2 (Fig. 4a). Although further studies with a large number of patients are required to confirm this observation, this exploratory investigation prompt us to test the potential effects of CNTD2 on cancer cell motility. For this purpose, confluent monolayers of A549 cells overexpressing CCNO or CNTD2 were scratched and wound closure determined 48 h later. A549 cells infected with empty vector were used as controls and reduced FBS media was used to suppress cell proliferation. As shown in Fig. 4b and c, migration of A549 cells overexpressing CNTD2 was significantly increased in comparison to control cells while CCNO did not significantly affect cell migration. Similarly, transwell assays demonstrated greater migration of A549 cells overexpressing CNTD2 to the lower chamber compared with control (Fig. 4d and e). Toward exploring potential mechanisms, we examined effects of CNTD2 upregulation on expression of epithelial-to-mesenchymal transition (EMT) markers. A549 cells overexpressing CNTD2 expressed significantly lower levels of E-cadherin but N-cadherin was slightly increased (Supplementary Fig. S5), indicating CNTD2 might influence the EMT.

### High levels of CNTD2 are associated with enhanced tumor growth *in vivo* and decreased overall survival of lung cancer patients

**Figure 5:**
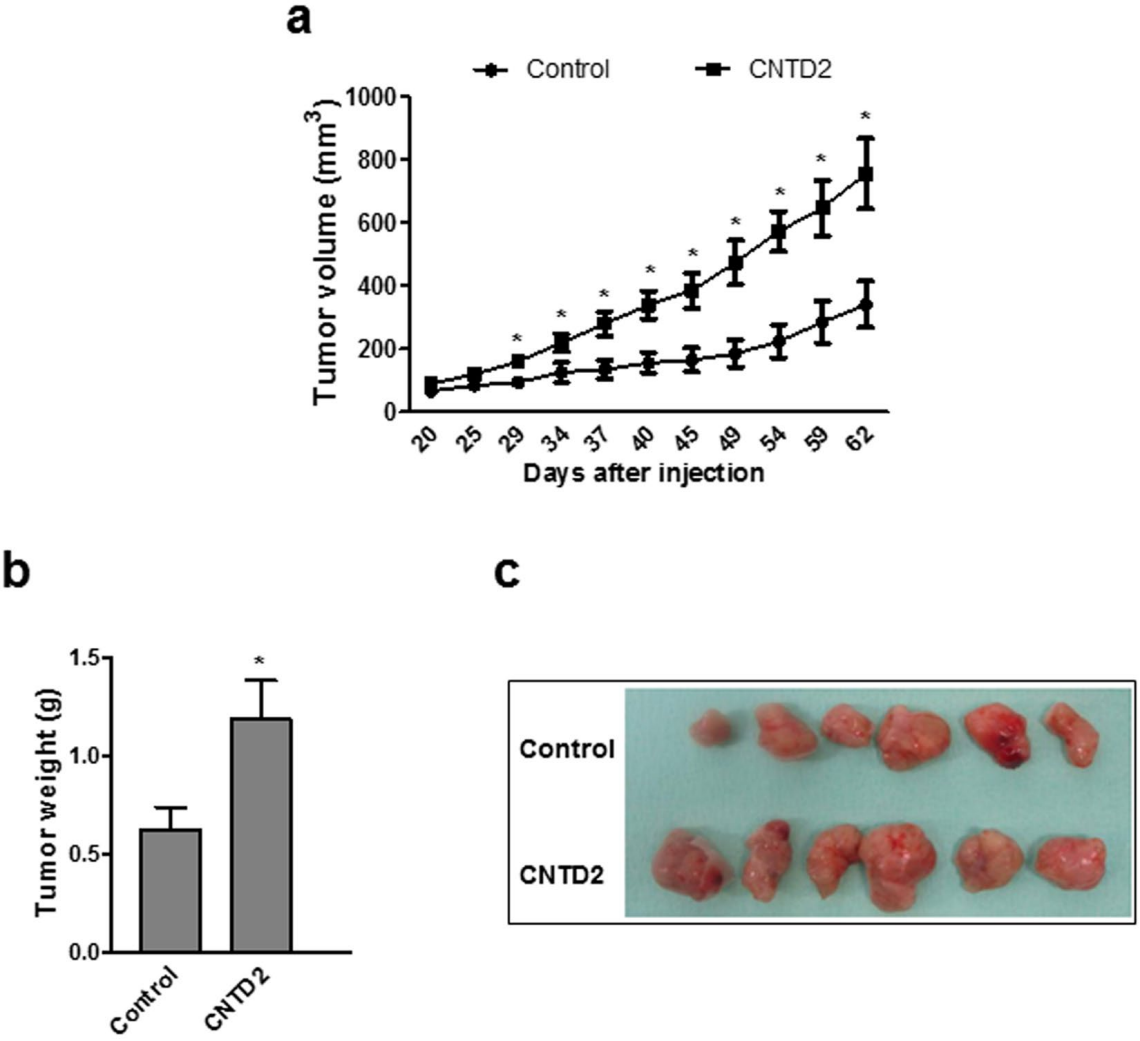
CNTD2 overexpression significantly enhances tumor growth *in vivo*. (**a**) The tumor volumes were measured at the indicated number of days after mice were transplanted with A549-empty vector (control) and A549-CNTD2 (n = 7). *P < 0.05, vs control, Mann-Whitney test. (**b**) At day 62, the tumors were resected and weighed. *P < 0.05, vs control, Mann-Whitney test. (**c**) Representative images of the tumors resected at day 62 obtained from A549-empty vector (control) and A549-CNTD2 cell-transplanted mice.

In order to investigate the effect of CNTD2 expression on lung cancer growth *in vivo*, we examined xenografts in nude mice formed by injection of A549 cells infected with empty vector (control) or overexpressing CNTD2. As shown in Fig. 5, A549 cells overexpressing CNTD2 formed faster growing tumors.

**Figure 6:**
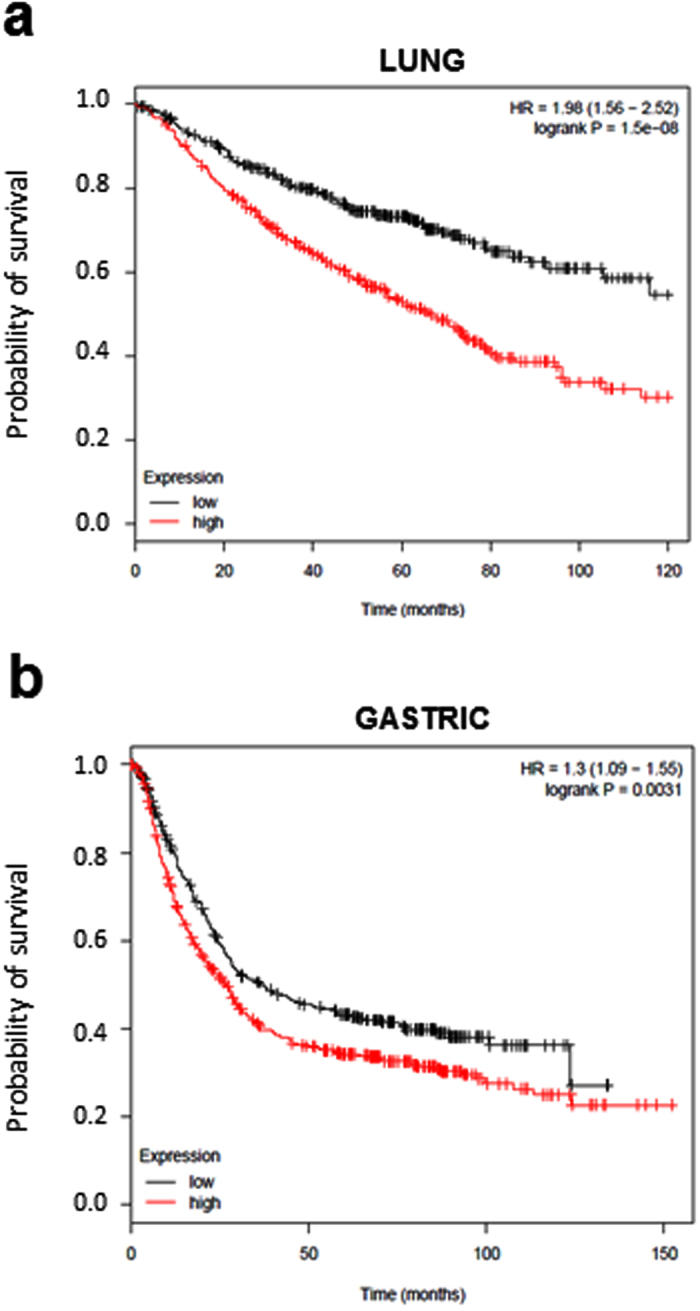
Prognostic value of CNTD2 on lung cancer survival. Kaplan-Meier plots were built using the Kaplan-Meier plotter software (http://kmplot.com/lung/). The overall survival of (**a**) lung cancer (n = 726) and (**b**) gastric cancer patients (n = 867) of all subtypes expressing different levels of CNTD2 is shown. Red colored lines represent the patients with high gene expression, while black colored lines represent patients with low gene expression. The hazard ratio (HR) with 95% confidence intervals, as well as the logrank P-values are shown.

Finally, the prognostic value of CNTD2 gene expression in a large set of lung cancer patients was assessed. We used Kaplan-Meier Plotter to compare overall survival of patients stratified according to levels of CNTD2 expression. High CNTD2 expression correlated with reduced overall survival (Fig. 6a). A similar analysis of ovarian, breast, and gastric cancer revealed that high CNTD2 expression also predicted shorter overall survival but only for gastric cancer (Fig. 6b).

## Discussion

The management of lung cancer remains a major challenge given that it is often diagnosed at an advanced stage of the disease that has a dismal prognosis. As the oncogenesis of lung cancer involves multiple molecular alterations, the development of new biomarkers would be helpful to guide treatment selection, as well as to avoid unnecessary toxicity and to improve the outcomes^5^. In the present study, we conducted an unbiased screening of the protein levels of eight orphan cyclins in lung cancer tissues, which revealed that some are deregulated and might be important factors in lung cancer development.

In our samples of FFPE tissues, we were unable to detect the expression of CCNG1, CCNG2, CNTD1 and SPY1 (Supplementary Fig. S2a). Interestingly, the Kaplan-Meier plots indicate that the overexpression of these cyclins is not associated with a worse prognosis of lung cancer patients (Supplementary Fig. S6a). On the contrary, high levels of CCNG1 were correlated with an increased overall survival of these patients (Supplementary Fig. S6a), pointing the high expression of CCNG1 as a good prognostic factor in lung cancer.

Moreover, according to the Kaplan-Meier Plotter database, CCNO upregulation is significantly associated with a reduced overall survival of lung cancer patients (Supplementary Fig. S6a), suggesting that CCNO is another orphan cyclin involved in the malignancy of the disease. However, we could not find a correlation between CCNO overexpression and the proliferation phenotype or the migration ability in A549 cells (Figs 3 and 4); indeed, although the upregulation of CCNO slightly increases the migration of A549 cells, no statistical significance was found in our experimental setting (Fig. 4). Taken together, these results indicate that CCNO might be implicated in processes other than the proliferation and migration of lung cancer cells. Accordingly, it was reported that CCNO is an important element for the physiology of lungs, and that mutations in the CCNO gene result in congenital lung disease affecting the multicilliated lung cells^19^.

In the present study, we found no statistical difference regarding the expression of CCNY in healthy and tumor tissues (Fig. 1b and c). In line with this observation, the expression of this protein does not correlate with the overall survival of lung cancer patients (Supplementary Fig. S6a), suggesting that CCNY might not play a role in lung cancer disease. On the other hand, the role of CCNY is clearly established in certain tumors, such as glioma^20^ and ovarian cancer^21^ indicating that its role in tumorigenesis might be tissue-specific. While previous reports have shown that CCNY is overexpressed in samples obtained from NSCLC patients^22^, we were unable to detect overexpression of CCNY in our samples (Fig. 1b and c). Since the work conducted by Yue *et al*.^22^ determined the levels of CCNY mRNA, while we measured the protein levels, these conflicting results may reflect the different readouts, in agreement with the observation that the protein expression in lung adenocarcinomas does not always correlate with the levels of the corresponding mRNA^16^, highlighting the importance of measuring the protein levels in this disease.

Our study detected that CCNI is upregulated at the protein level in human samples of NSCLC (Fig. 1b and c). Moreover, the overexpression of this cyclin increased the proliferation of A549 cells, as suggested by the results of the MTT assay (Fig. 3b) and ki-67 staining (Fig. 3c and d), unveiling a role for this cyclin in lung cancer cell proliferation. Interestingly, CCNI overexpression was also associated with worse response to anticancer agents (Supplementary Fig. S4), which is in agreement with the observation that CCNI promotes resistance to cisplatin in cervical cancer^23^. In our study, the resistance provided by CCNI seems to be unrelated with the chemical structure of the drugs, and further studies are now warranted to clarify whether multidrug resistance proteins might be implicated in this response. Moreover, CCNI was linked to angiogenesis in ovarian^24^ and breast cancers^25^, suggesting that its role in cancer may go beyond cell proliferation. Remarkably, the Kaplan-Meier analysis did not find a correlation between CCNI overexpression and the overall survival of lung cancer patients of all histopathological types, but statistical significance could be found when the same analysis was performed only in patients with adenocarcinoma (Supplementary Fig. S6b). Given the variability in lung cancers, it is plausible that CCNI may have a relevant role in some lung cancer subtypes; indeed, the two adenocarcinoma cell lines analyzed display significant differences regarding CCNI expression relative to normal human fibroblasts (Fig. 1a).

Our results show that CNTD2 is important for both the proliferation and migration of A549 cells (Figs 3 and 4), and suggest that it may enhance lung cancer cell migration by promoting the EMT (Supplementary Fig. S5). The association between CNTD2 expression and a more aggressive phenotype was further corroborated by the results obtained *in vivo* (Fig. 5), as well as by the dramatic correlation between high levels of CNTD2 and a decreased overall survival of a large set of lung cancer patients (Fig. 6).

Interestingly, our results represent a new piece of information that has remained undetected by the analysis of large-scale genomic datasets screenings, such as the one provided by the web-based resource cBioPortal for Cancer Genomics (http://cbioportal.org ^26^), which reported a low frequency of CNTD2 alterations in cancer. Likewise, the website-based software Kaplan-Meier Plotter (http://kmplot.com/lung/ ^27^) indicates similar levels of mRNA of CNTD2 in normal and lung cancer tissues. On the other hand, the Oncomine database (http://www.oncomine.org) demonstrates that high levels of CNTD2 mRNA are present in lung cancer tissues (Supplementary Fig. S7a), in line with our findings (Fig. 1b and c). Although we did not measure the levels of mRNA in our samples, these conflicting evidences reinforce the need to monitor the final product of gene expression, rather than the intermediate.

Strikingly, we were unable to detect CNTD2 protein expression in lung cancer cell lines (Supplementary Fig. S2a). According to the Expression Atlas database (https://www.ebi.ac.uk), CNTD2 is expressed in a few lung cancer cell lines (Supplementary Fig. S7b). While A549 cells present low levels of mRNA, the NCI-H1395 cells are among those showing higher levels of CNTD2 mRNA (Supplementary Fig. S7b), but the level of protein expression is still not appreciable to allow the antibody detection (Supplementary Fig. S2a). Such observations may suggest that CNTD2 expression is differentially regulated in cultured cells as compared with tissues.

CNTD2 is probably the less characterized of the orphan cyclins. While little is known about the cellular functions of CNTD2, its predominant nuclear location (Fig. 2) may implicate CNTD2 in transcription regulation as described for other cyclins. On the other hand, its name (CNTD2 stands for Cyclin N-terminal domain-containing protein 2) shows that this protein contains a cyclin box domain and, hence, that it might interact with some CDK. We have tried to detect the partners of CNTD2 by immunoprecipitation coupled to mass spectrometry, but we have not yet been able to identify any CNTD2-associated CDK (Ribeiro *et al*., unpublished observations). Therefore, whether this still orphan cyclin performs its nuclear function alone or in association with a CDK remains to be elucidated.

Here we report that CNTD2 is upregulated in human lung cancer tissues and correlates with worse prognosis. Our findings suggest that CNTD2 is an oncogenic driver in lung cancer, and further studies that may shed some light on the physiological and pathological role of CNTD2 are now warranted. Interestingly, CNTD2 does not seem to be expressed in normal tissues^28^, suggesting that this cyclin may represent a specific target for future therapies. The identification of CNTD2 interactors would possibly pave the way for the development of new therapeutic strategies targeting CNTD2.

## Methods

### Clinical samples

Lung cancer tissue samples and adjacent non-tumor lung tissue were obtained from 43 patients diagnosed with NSCLC at the Hospital Universitari General de Catalunya and Hospital Universitari Sagrat Cor. For inclusion in this study, a patient must have had a diagnosis of primary lung cancer and did not receive chemotherapy or radiation therapy before surgery. The samples were fixed in formaldehyde and embedded in paraffin (FFPE) according to routine procedures at these hospitals. Histologically, the tumors were classified according to the 2015 World Health Organization histologic classification of lung cancers and were reviewed by the same pathologist. Among the 43 samples, 28 were adenocarcinomas and 15 were squamous cell carcinomas. The tumors were staged using the TNM staging system and the patients’ characteristics are summarized in Supplementary Table S1. This study was approved by the Comitè Étic de Investigació Clínica (CEIC) de idcsalud Hospital General de Catalunya and carried out in accordance with the approved guidelines. The requirement to obtain informed consent was waived by the Ethics Committee because of the retrospective nature of the study, but an informed consent was obtained from living patients.

### Cell lines and Reagents

The human lung adenocarcinoma cell lines A549 (purchased in 2014 from Sigma-Aldrich) and NCI-H1395 (purchased in 2016 from ATCC) were cultured in Dulbecco’s Modified Eagle Medium (DMEM; Sigma-Aldrich). The human lung fibroblast cell line MRC-5 was purchased from ATCC in 2016 and cultured in Eagle’s Minimum Essential Medium (ATCC). Cells were used for no more than 5 passages after thawing. The media were supplemented with 10% heat-inactivated fetal bovine serum (FBS; Sigma-Aldrich) and 1% penicillin/streptomycin (Sigma-Aldrich). The cells were kept at 37 °C in a humidified incubator with 5% CO_2_. Cisplatin, paclitaxel and gefitinib were purchased from Sigma-Aldrich. Mycoplasma contamination was monitored periodically.

### Protein extraction from FFPE tissues

Sample blocks consisting of >80% tumor tissue were selected along with paired normal controls. Following standard methods for protein extraction^29, 30^, tissue sections (10 μm) were deparaffinized and rehydrated. After incubation on ice with 100 µl of lysis buffer (20 mM Tris-HCl pH = 8.8, 200 mM DTT, 2% SDS) for 5 min, and placed at 100 °C for 20 min. Then, samples were placed on a thermomixer for 2 h, at 80 °C and 750 rpm. Afterwards, the samples were centrifuged at 14,000 rpm for 15 min at 4 °C, and the supernatant was collected for subsequent analysis.

### Protein extraction from human cell lines

Cultured human cell lines were washed with ice-cold phosphate-buffered saline (PBS) and placed on lysis buffer, containing 20 mM TRIS, 5 mM EDTA, 1% NP40 (IGEPAL CA-630), 150 mM NaCl, pH 7.4, supplemented with Pierce Phosphatase Inhibitor Mini tablets (#88667, Thermo Fisher Scientific) and with Pierce Protease Inhibitor tablets (#88266, Thermo Fisher Scientific). Extracts were sonicated and centrifuged at 14,000 rpm for 15 min at 4 °C, and the supernatants collected.

### Western blot analysis

Protein concentration was quantified by Bradford assay (BioRad). Gel samples were denatured at 90 °C for 5 min and 25 µg of total protein was separated by 10% SDS-PAGE and transferred to PVDF membranes (Immobilon-P, Millipore). All the information regarding the commercial primary antibodies used is indicated in Supplementary Table S2. After blocking, the membranes were incubated with the primary antibody in TBS-T + 5% non-fat milk overnight at 4 °C. After extensive washing, membranes were incubated with horseradish peroxidase (HRP) conjugated anti-mouse, -rabbit, and -goat IgG secondary antibodies (Jackson Laboratories) for 1 h at room temperature. After additional washes, the membranes were developed using Luminata Forte Western HRP Substrate (Millipore) following manufacturer’s instructions, and images were taken with GeneSnap (Syngene). The density of the bands was analyzed using the Image Studio Lite (Li-Cor) software.

### Validation of antibodies

Human orphan cyclins were cloned in pGBKT7 vector (Clontech) as Gal4 DNA-binding domain (DNA-BD) fusion proteins, which were expressed in the yeast strain AH109. Western blot analysis against both the Gal4 DNA-BD and the corresponding cyclin confirmed the validity of the antibodies (Supplementary Fig. S1).

### Immunodetection

Immunohistochemical staining of FFPE samples was performed on 4 µm sections using a BenchMark Ultra (Ventana Medical Systems Inc.). The following polyclonal antibodies were used: CNTD2 (ab179781, Abcam) at a dilution of 1:100; CCNO (ab47682, Abcam) at a dilution of 1:100; CCNI (H-279, Santa Cruz Biotechnology) at a dilution of 1:50; and CCNY (ab114086, Abcam) at a dilution of 1:100. The sections were introduced in the stainer and automatically deparaffinized with the reactive EZ-PREP and treated with CC2 cell conditioning 6.1 for antigen retrieval for 30 min at 95 °C. After incubation with antibodies, immunoreactivity was detected with Kit uView DAB with diaminobenzidine (DAB) as a chromogen and counterstained with hematoxylin. Appropriate controls were included. For protein staining, two categories were recorded: negative and positive; only convincing staining was designated as positive.

For Ki-67 analysis, A549 infected cells were seeded on poly-L-lysine coated coverslips in 24 well plates at a density of 25,000 cells per well and, after 24 h, the cells were fixed in cold 4% paraformaldehyde at 4 °C for 15 min. Afterwards, the cells were blocked with PBS containing 0.1% Triton and 10% horse serum for 1 h, followed by incubation with Ki-67 antibody (556003, BD Pharmingen) diluted at 1:200 for 1 h at room temperature. The coverslips were washed several times with PBS and incubated for 1 hour at room temperature with anti-mouse fluorescent secondary antibody at 1:1,000 dilution in the dark. After additional washes with PBS, the nuclei were counterstained with Hoechst 33258 (14530, Sigma-Aldrich) and the slides coverslipped. Image analysis was performed using NIS-Elements AR 4.13.04 software. Between 50 and 70 cells were analyzed per condition.

### Viral cloning and transduction

To engineer lentiviral constructs expressing the cyclins under investigation, human cDNA of CCNO, CCNI and CCNY were amplified from HT-29 cells with a C-terminal FLAG tag. CNTD2 was purchased as a gBlock (IDT) based on Ensembl sequence ENST00000430325.6. The primer sequences used in this study are indicated in the Supplementary Table S3.

All the sequences were cloned into pWPI lentiviral expression vector (#12254, Addgene) at the *Pme*I restriction site. For lentivirus production, 45 μg of lentiviral expression vector were cotransfected with 12.9 μg of pMD2.G and 29.1 μg of psPAX2 into HEK293-T cells in 10 cm plates using calcium phosphate. The virus-containing supernatant was collected at 24 h and 48 h post-transfection, and concentrated using the Sartorius VS2042 Vivaspin 20 concentrator (Sartorius) and the viral titer determined. For overexpression studies, A549 cells were infected with 10 MOI of lentivirus.

### Cell viability and colony formation assays

Cell viability was evaluated by MTT assay. Cells were grown in 48 well plates at a density of 1,500 cells per well. Seventy-two hours later, the cells were infected, and after 5 days the cells were incubated with MTT solution (Sigma) for 1 h, at 37 °C. Formazan crystals were dissolved in DMSO and quantitated by measuring the absorbance in a Synergy HT plate reader at 570 nm. The reduction of MTT is expressed as percentage of the absorbance value obtained in the control which was considered 100%. For experiments using anti-cancer agents, cells were seeded in 24 well plates at a density of 60,000 cells per well, and after 24 h the cells were treated with cisplatin, paclitaxel, or gefitinib. MTT assay was performed as described above 72 h later.

For colony formation assay, A549 cells were seeded into 6 well plates at a density of 100 cells per well and cultured for two weeks. Colonies were fixed with 100% cold methanol, washed with PBS and stained with 0.1% crystal violet for 30 min at room temperature. The number of colonies was determined and survival calculated as a percentage of the control.

### Wound healing and transwell migration assays

For wound healing assay, cells were seeded into 24 well plates at a density of 2.5 × 10^5^ of cells per well and grown to confluency. The cell monolayer was scratched with a 10 µl pipette tip, washed with PBS to remove detached cells from the plates, and incubated in DMEM containing 0.5% FBS. Images were acquired at 0and 48 h under a 4× magnification and the distance between one side of the wound and the other was measured using ImageJ. Cell migration was determined by calculating the wound closure, which corresponds to the difference between the size of the wound at 0 h and at the indicated time point, and it is expressed as µm.

For migration assays, cells were seeded into the upper chamber of an 8 µm pore size insert (Corning) in an uncoated 24 well plate at a density of 5 × 10^4^ cells per well. The cells were cultured in DMEM containing 0.5% FBS, and allowed to migrate to the lower compartment containing DMEM 10% FBS for 18 h. The nonmigratory cells on the surface of the upper chamber were removed with a cotton tip, and the migratory cells attached to the lower membrane surface were fixed with cold methanol for 15 min at 4 °C and stained with 0.1% crystal violet for 30 min at room temperature. Cells were imaged under a 10× objective. To estimate the number of cells in the lower compartment, the crystal violet dye was dissolved using acetic acid at 10% and absorbance was measured at 595 nm. The crystal violet staining intensity is expressed as percentage of the absorbance value obtained in the control which was considered 100%.

### Tumor xenografts

Athymic nude mice male (Crl:NU-Foxn1nu) (n = 7) at 5-weeks-old male (Envigo, Italy) of age were subcutaneously injected in each flank with a total of 3 × 10^6^ A549-empty vector or A549-CNTD2-transfected cells soaked in 100 µL of Matrigel (BD Biosciences). Tumor growth was monitored by measuring tumor width (W) and length (L) until mice were killed, 62 days after injection. After allowing them to grow for several weeks, tumor volume (mm^3^) was estimated from the formula V = π/(6 × L × W2) and at the mice sacrifice the tumors were dissected out and weighed (g). All mouse experiments were approved by the IDIBELL Animal Care Committee and the methods were carried out in accordance with the approved guidelines.

### Kaplan-Meier analysis

For survival analyses, the overall survival of patients divided into low and high expression groups according to the median expression of the gene of interest was presented as Kaplan-Meier plots. The analysis was conducted as described (http://kmplot.com/lung/) selecting only patients with surgical margins negative and tested for significance by two-tailed log rank t test^27^.

### Statistical analysis

Unless otherwise stated, data are represented as the mean ± standard error of the mean (SEM) with P-values: ***P < 0.001; **P < 0.01; *P < 0.05. Statistical significance was determined using the Mann-Whitney test, except for the expression ratio between tumor and normal tissues which was analyzed using the Wilcoxon test. Statistical analyses were conducted using GraphPad Prism 5 and the Statistical Package for the Social Sciences (SPSS) 21.

## Electronic supplementary material

Supplementary figures and tables
