# Supplementary material for: A systematic analysis of orphan cyclins reveals CNTD2 as a new oncogenic driver in lung cancer
Source: Sci Rep. 2017 Aug 31;7:10228. doi: 10.1038/s41598-017-10770-8 (PMC5579190; doi:10.1038/s41598-017-10770-8)
Supplement: Supplementary file 1 — Supplementary figures and tables [file 41598_2017_10770_MOESM1_ESM.pdf]

A systematic analysis of orphan cyclins reveals CNTD2 as a new oncogenic driver in lung cancer

Gasa L[&], Sanchez-Botet A[&], Quandt E, Hernández-Ortega S, Jiménez J, Carrasco-García MA, Simonetti S, Kron SJ, Ribeiro MP[*], Nadal E, Villanueva A, Clotet J[*]

[&]Contributed equally
*Corresponding authors

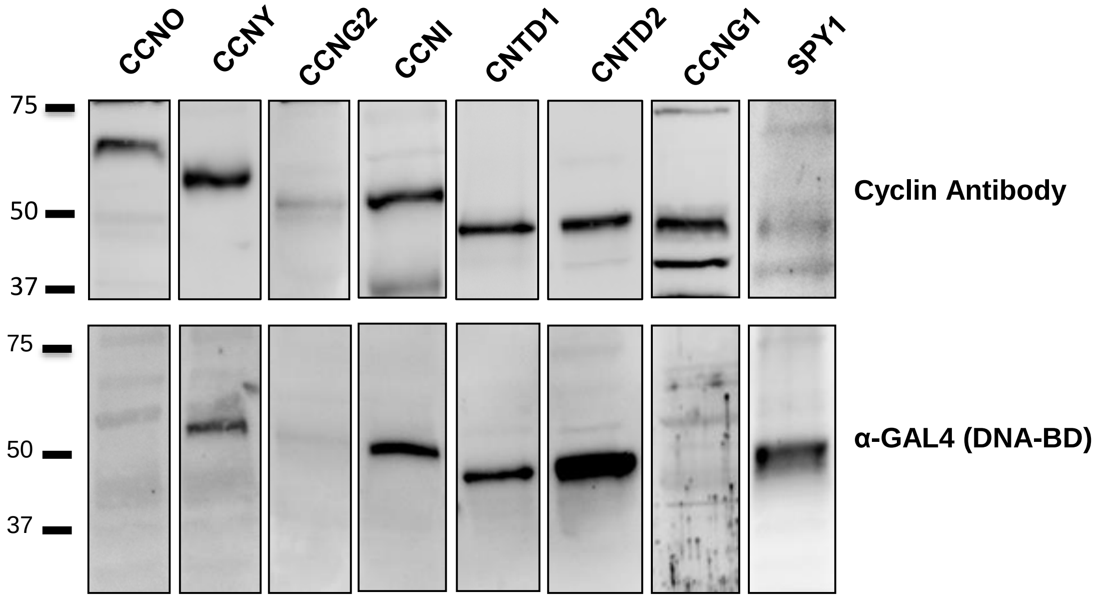

Supplementary Figure S1. Validation of the cyclins antibodies. The cyclins were cloned into pGBKT7 vector as Gal4 DNA-binding domain (DNA-BD) fusion proteins and expressed in the yeast strain AH109. The cyclins expression was detected using an anti-Gal4 antibody (bottom panel) and, afterwards, each lane was reprobed with the corresponding commercial antibody (top panel).

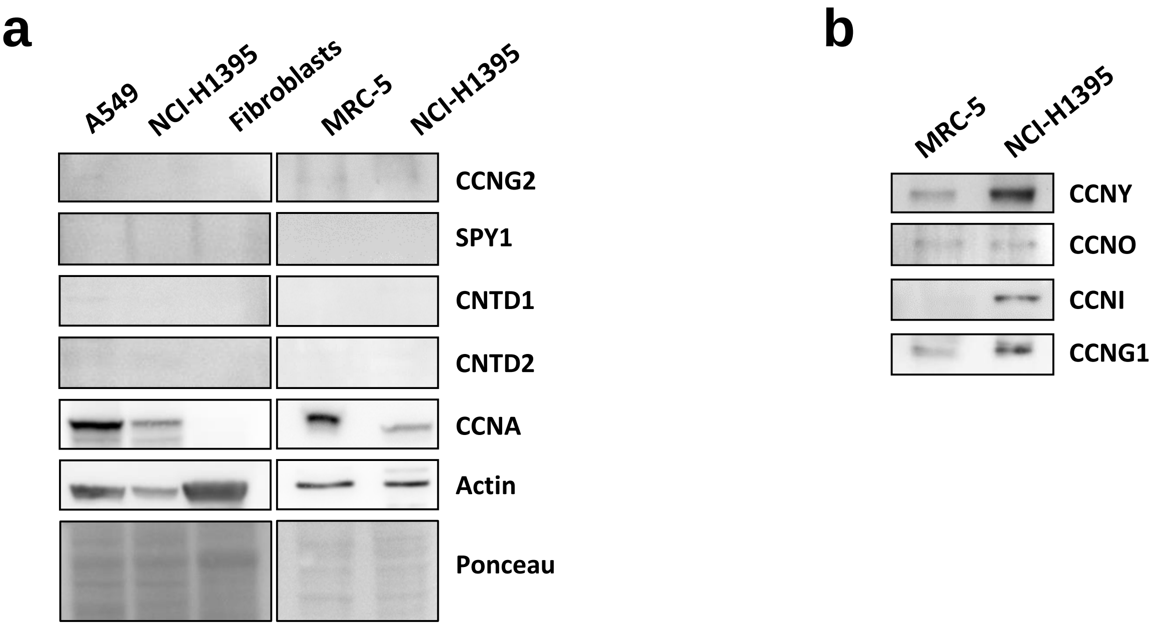

Supplementary Figure S2. Orphan cyclins expression in human lung cell lines. The protein expression level of orphan cyclins was evaluated by western blot in lung adenocarcinoma cell lines, A549, NCI-H1395, a primary culture of fibroblasts, and in the human lung fibroblast cell line MRC-5. (a) The expression of CNTD1, CNTD2, CCNG2 and SPY1 was undetectable in all the cells tested. (b) Representative images of the western blot analysis in NCI-H1395 and in the MRC-5 cells. The corresponding loading control is shown in (a).

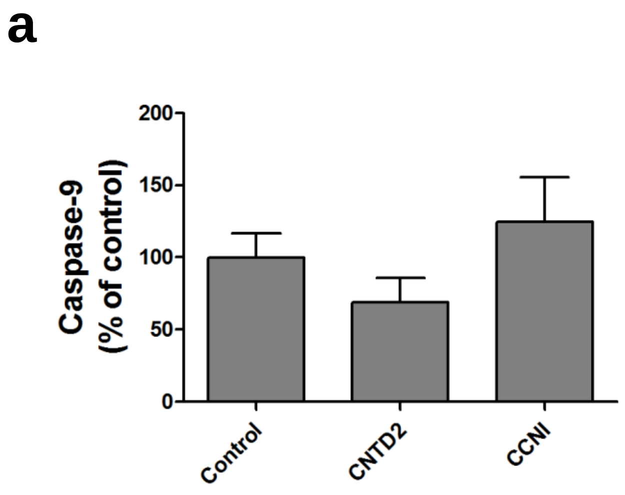

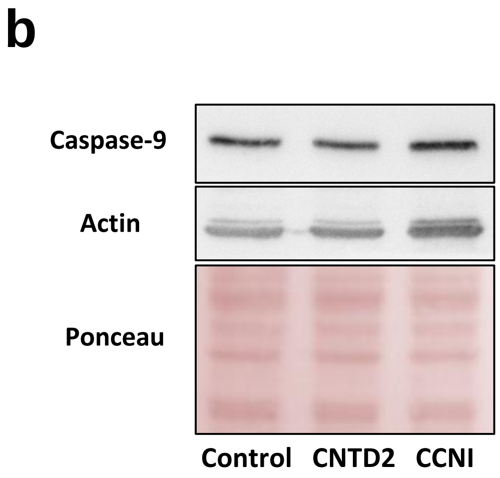

Supplementary Figure S3. CNTD2 and CCNI overexpression does not promote A549 lung cancer cells apoptosis. The expression of Caspase-9 was monitored in A549 cells infected with empty lentiviral vector (control) or with the indicating cyclin-overexpressing construct by western blot. (a) After normalization with Ponceau, the overexpression effect of the cyclins tested on caspase-9 was quantitated. Columns represent the mean ± SEM of five independent experiments, Mann-Whitney test. (b) Representative images of the western blot analysis.

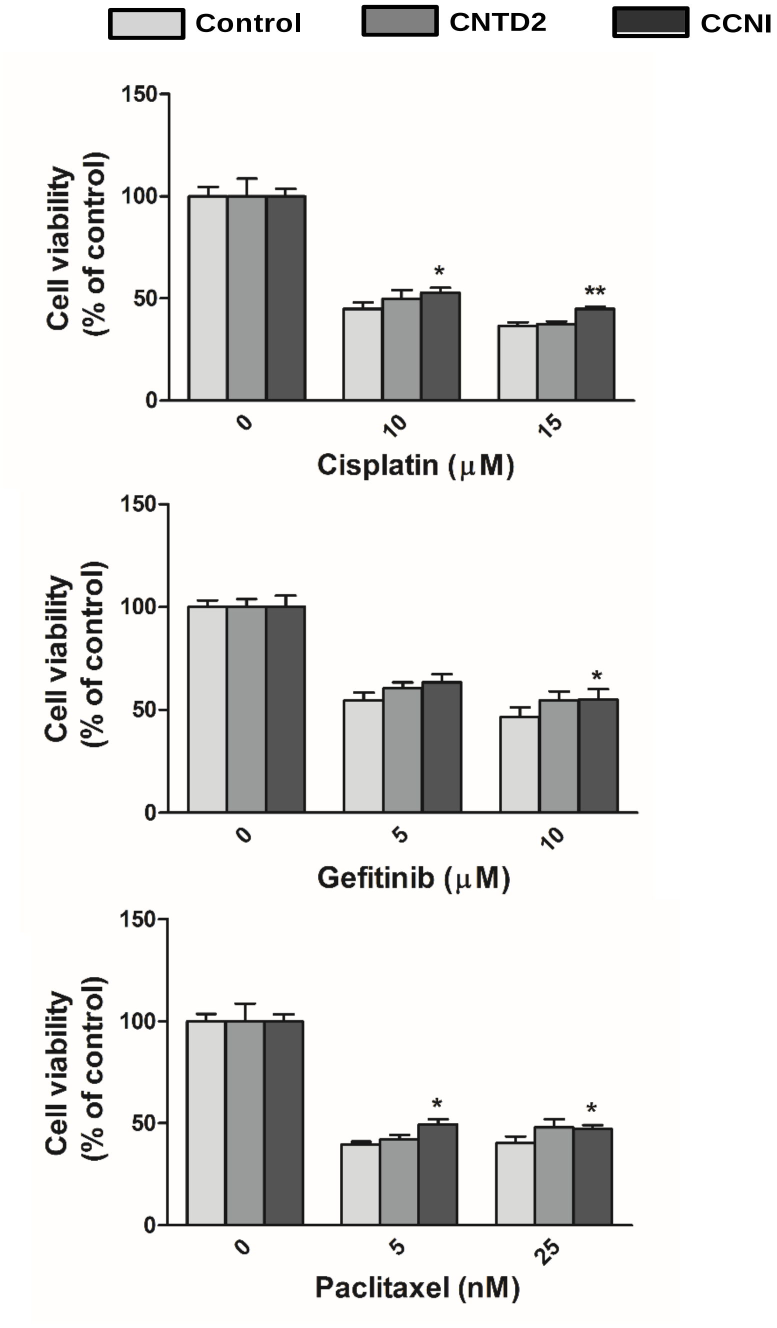

Supplementary Figure S4. CCNI overexpression is associated with increased lung cancer resistance against antitumoral agents. A549 cells infected with empty lentiviral vector (control) or with the cyclin-overexpressing construct for CCNI and CNTD2 were treated with the indicated concentrations of cisplatin, gefitininb and paclitaxel. Cell viability was estimated by the MTT assay 72 h later and is expressed as percentage of untreated cells. Columns represent the mean ± SEM of four independent experiments performed in quadruplicates. *P < 0.05, **P < 0.01 vs control under the same drug treatment, Mann-Whitney test.

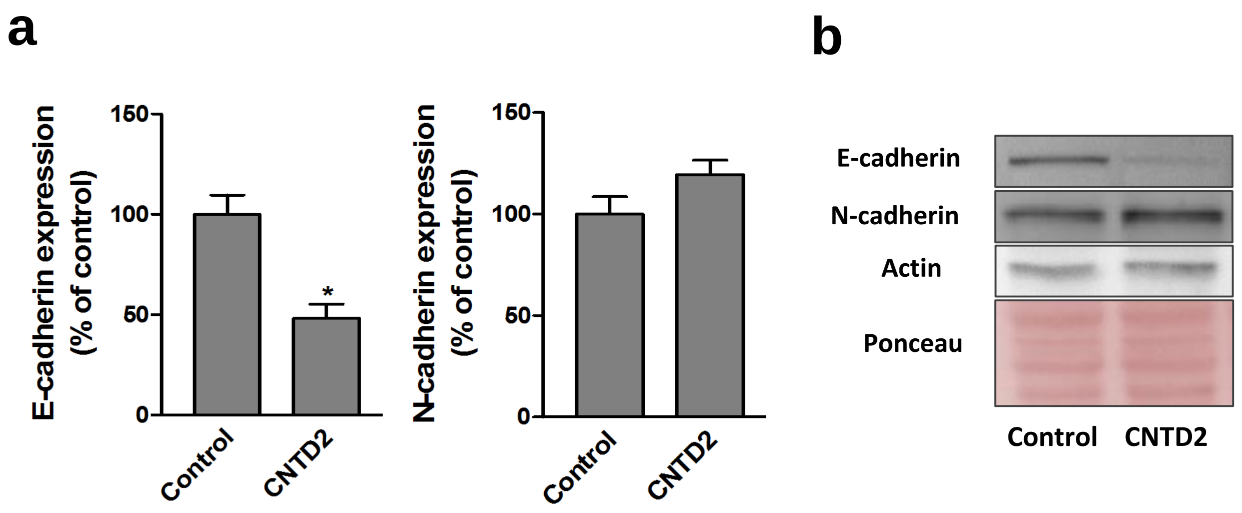

Supplementary Figure S5. CNTD2 promotes the EMT. The expression of E-cadherin and N-cadherin was monitored in A549 cells infected with empty lentiviral vector (control) or with CNTD2-overexpressing construct by western blot. (a) The effect of CNTD2 overexpression on cadherin expression was quantitated after normalization with Ponceau and is expressed as percentage of control. Columns represent the mean ± SEM of twelve independent experiments. *P < 0.05 vs control, Mann-Whitney test. (b) Representative images of the western blot analysis.

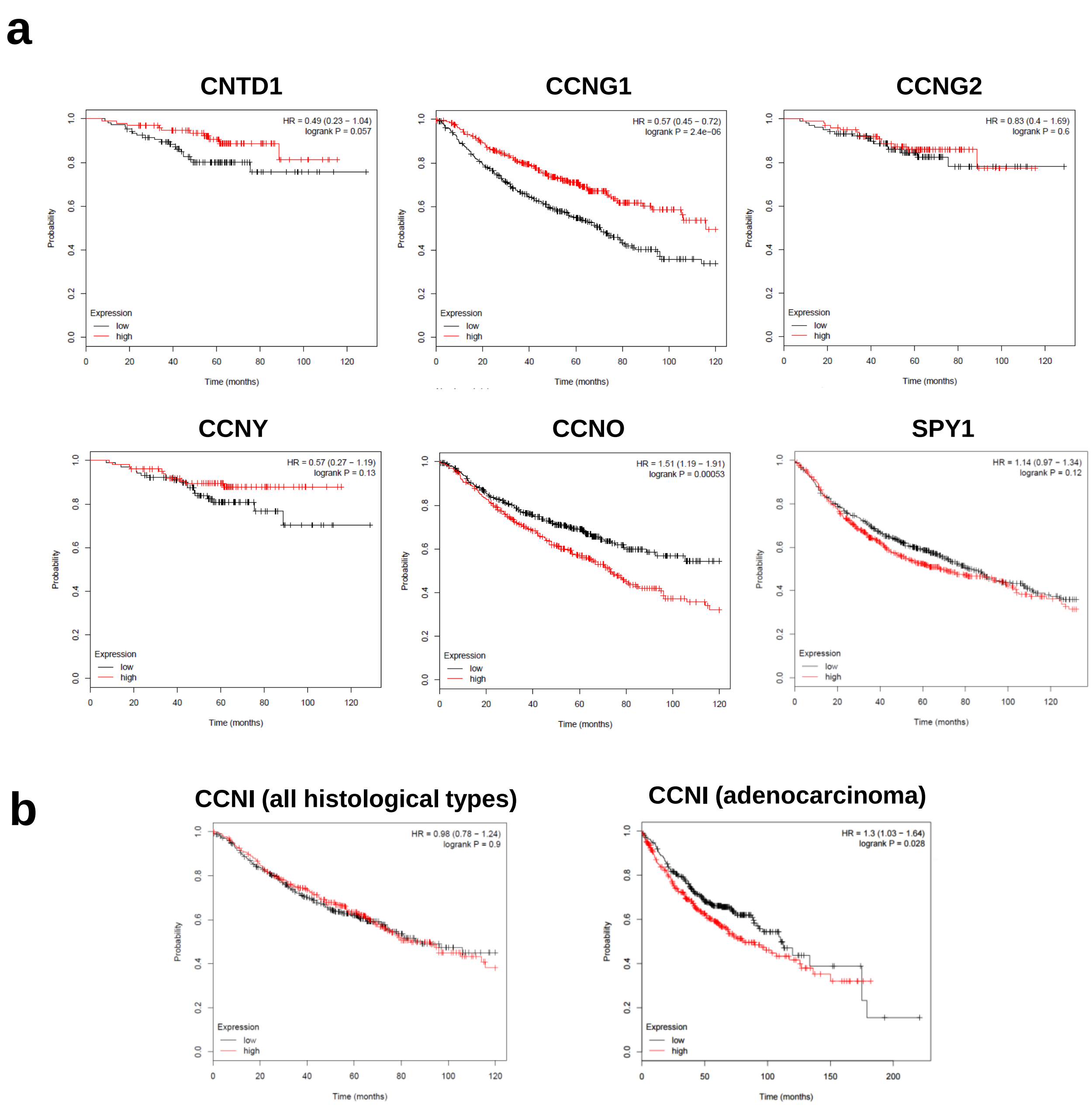

Supplementary Figure S6. Prognostic value of orphan cyclins on the overall survival of lung cancer patients. (a) Kaplan-Meier plots representing the overall survival of lung cancer patients of all histological types (n=726) expressing different levels of the indicated cyclins. (b) Kaplan-Meier plots showing the correlation between different expression levels of CCNI and the overall survival of lung cancer patients of all histological types (left panel, n=1926) and adenocarcinoma (right panel, n=867). Red colored lines represent the patients with high gene expression, while black colored lines represent patients with low gene expression. The hazard ratio (HR) with 95% confidence intervals, as well as the logrank P-values are shown.

Figure S7
Gasa et al

a

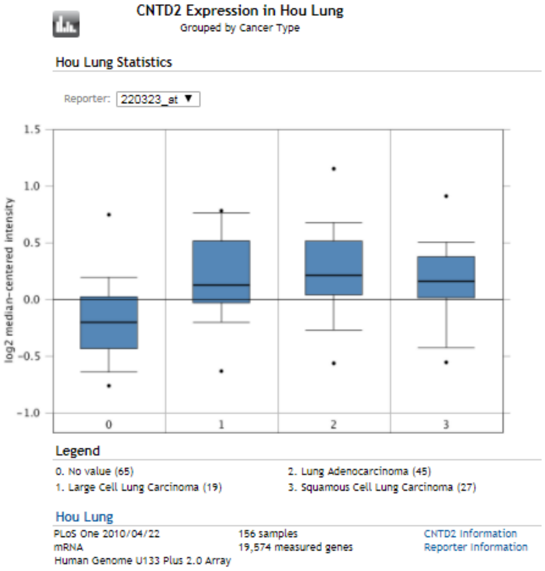

b

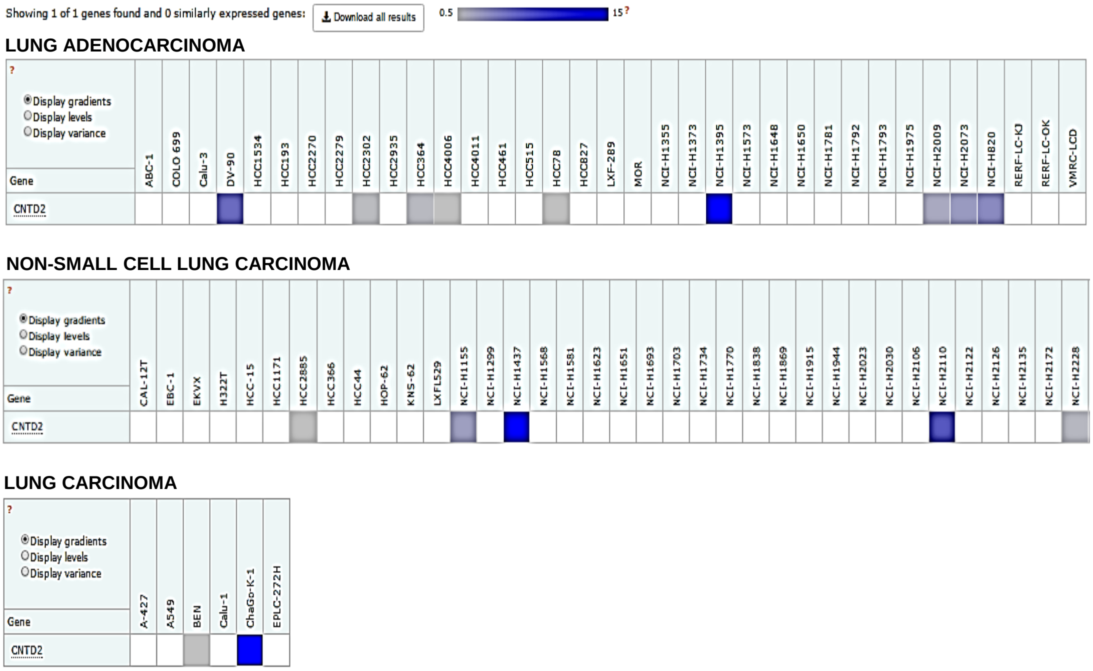

Supplementary Figure S7. Differential CNTD2 expression in human lung cancer tissues and cancer cell lines. (a) Levels of CNTD2 mRNA in human lung cancer samples of different histological types (65 normal samples, 19 large carcinoma samples, 45 lung adenocarcinoma samples and 27 squamous cell carcinoma samples) according to the Oncomine database (http://www.oncomine.org). (b) CNTD2 mRNA levels in different human lung cancer cell lines according to the Expression Atlas website (https://www.ebi.ac.uk).

		Number (%)
Histologic Type		
	Adenocarcinoma	28 (65.1%)
	Squamous cell carcinoma	15 (34.9%)
Stage		
	I	25 (58.1%)
	II	12 (27.9%)
	III	3 (7%)
	IV	3 (7%)
Sex		
	Female	14 (32.6%)
	Male	29 (67,4%)
Age (years)		65,9 (60 -74)
Smoking history		
	Never smoke	6 (14%)
	Ever smoke	34 (79%)
	-	3 (7%)
Other tumors		
	Never	38 (88.4%)
	Ever	5 (11.6%)

Supplementary Table S1. Clinicopathologic features of the subjects included in the study (n=43).

Cat. No	Antibody	Species	Dilution	Company
2956	GFP	Rabbit	1:500	Cell Signaling
ab179781	CNTD2	Rabbit	1:2,000	Abcam
ab47682	CCNO	Rabbit	1:500	Abcam
NB100-2521	SPY1	Rabbit	1:550	Novus Biologicals
ab114086	CCNY	Rabbit	1:1,000	Abcam
ab126998	CNTD1	Rabbit	1:6,000	Abcam
sc-320	CCNG1	Rabbit	1:200	Santa Cruz Biotechnology
sc-7266	CCNG2	Goat	1:500	Santa Cruz Biotechnology
sc-5547	CCNI	Rabbit	1:400	Santa Cruz Biotechnology
F3165	FLAG	Mouse	1:500	Sigma-Aldrich
610921	N-Cadherin	Mouse	1:1,000	BD Biosciences
sc-73548	Caspase-9	Mouse	1:200	Santa Cruz Biotechnology
sc-1500	E-Cadherin	Goat	1:200	Santa Cruz Biotechnology
A5316	β-Actin	Mouse	1:1,000	Sigma-Aldrich

Supplementary Table S2. List of antibodies and corresponding working dilutions.

	FORWARD	REVERSE
**CCNO**	GCCTCGAGGTTTAAACATGG TGACCCCCTGTCCCACCAGC CCCTCGAG	GCCCGTAGTTTAAACCTACTTATCG TCGTCATCCTTGTAATCTTTCGAGCT CGGGGGCAGGCTGCACTTCTCGC
**CNTD2**	GCCTCGAGGTTTAAACATGC TGGTGAGAGGCAGGGAC	GCCCGTAGTTTAAACCTACTTATCG TCGTCATCCTTGTAATCATAATTGTC TCTCATTC
**CCNI**	GCCTCGAGGTTTAAACATGA AGTTTCCAGGGCCTTTG	GCCCGTAGTTTAAACCTACTTATCG TCGTCATCCTTGTAATCCATGACAG AAACAGGCTG
**CCNY**	GCCTCGAGGTTTAAACATGG GGAACACTACCTCG	GCCCGTAGTTTAAACCTACTTATCG TCGTCATCCTTGTAATCAGAGATGAT GGCTGGGG

Supplementary Table S3. Sequences of the primers used.
